# Limb-specific thalamocortical tracts are impaired differently in hemiplegic and diplegic subtypes of cerebral palsy

**DOI:** 10.1093/cercor/bhad279

**Published:** 2023-08-18

**Authors:** Julia Jaatela, Dogu Baran Aydogan, Timo Nurmi, Jaakko Vallinoja, Helena Mäenpää, Harri Piitulainen

**Affiliations:** Department of Neuroscience and Biomedical Engineering, Aalto University School of Science, FI-02150 Espoo, Finland; Department of Neuroscience and Biomedical Engineering, Aalto University School of Science, FI-02150 Espoo, Finland; A. I. Virtanen Institute for Molecular Sciences, University of Eastern Finland, FI-70211 Kuopio, Finland; Department of Neuroscience and Biomedical Engineering, Aalto University School of Science, FI-02150 Espoo, Finland; Faculty of Sport and Health Sciences, University of Jyväskylä, FI-40014 Jyväskylä, Finland; Department of Neuroscience and Biomedical Engineering, Aalto University School of Science, FI-02150 Espoo, Finland; Pediatric Neurology, New Children’s Hospital, Helsinki University Hospital, FI-00029 Helsinki, Finland; Department of Neuroscience and Biomedical Engineering, Aalto University School of Science, FI-02150 Espoo, Finland; Faculty of Sport and Health Sciences, University of Jyväskylä, FI-40014 Jyväskylä, Finland; Pediatric Neurology, New Children’s Hospital, Helsinki University Hospital, FI-00029 Helsinki, Finland; Aalto NeuroImaging, Aalto University, FI-02150 Espoo, Finland

**Keywords:** diffusion MRI, fMRI-seeding, sensorimotor performance, stability, tractography

## Abstract

Thalamocortical pathways are considered crucial in the sensorimotor functioning of children with cerebral palsy (CP). However, previous research has been limited by non-specific tractography seeding and the lack of comparison between different CP subtypes. We compared *limb-specific* thalamocortical tracts between children with hemiplegic (HP, *N* = 15) or diplegic (DP, *N* = 10) CP and typically developed peers (*N* = 19). The cortical seed-points for the upper and lower extremities were selected (i) manually based on anatomical landmarks or (ii) using functional magnetic resonance imaging (fMRI) activations following proprioceptive-limb stimulation. Correlations were investigated between tract structure (mean diffusivity, MD; fractional anisotropy, FA; apparent fiber density, AFD) and sensorimotor performance (hand skill and postural stability). Compared to controls, our results revealed increased MD in both upper and lower limb thalamocortical tracts in the non-dominant hemisphere in HP and bilaterally in DP subgroup. MD was strongly lateralized in participants with hemiplegia, while AFD seemed lateralized only in controls. fMRI-based tractography results were comparable. The correlation analysis indicated an association between the white matter structure and sensorimotor performance. These findings suggest distinct impairment of functionally relevant thalamocortical pathways in HP and DP subtypes. Thus, the organization of thalamocortical white matter tracts may offer valuable guidance for targeted, life-long rehabilitation in children with CP.

## Introduction

Cerebral palsy (CP) is the most common motor disorder diagnosed in childhood, with a prevalence of 2–3 cases per 1,000 livebirths (Hagberg et al. 2001; Yeargin-Allsopp et al. 2008). CP is caused by damage or malformation in the developing brain and leads to challenges in motor control. The most common clinical symptom is spasticity, which typically affects either one side of the body (hemiplegia) or both sides, prominently the lower limbs (diplegia) (Bax et al. 2005). Despite the common CP diagnosis, the underlying changes in the brain structure can be variable, and lead to heterogeneous clinical presentations. To increase the benefits of life-long rehabilitation, it is essential to deepen our understanding of how different pathways in the brain contribute to sensorimotor performance.

The development of diffusion-weighted magnetic resonance imaging (dMRI) has provided evidence of widespread white matter involvement in the neuropathogenesis of CP (for a review see Scheck et al. 2012; Mailleux et al. 2020a). One of the major pathways affected is the ascending thalamocortical tract carrying tactile, proprioceptive, and kinesthetic information from the periphery to the primary sensorimotor (SMI) cortices (Hoon et al. 2002; Thomas et al. 2005; Nagae et al. 2007). Several studies in CP have reported that the fractional anisotropy (FA) of these ascending pathways is reduced whereas the mean diffusivity (MD) is increased (Trivedi et al. 2010; Pannek et al. 2014; Papadelis et al. 2014, 2018, 2019; Lennartsson et al. 2015; Ballester-Plané et al. 2017; Kuczynski et al. 2017). The lower FA and higher MD of the thalamocortical projections suggest reduced myelination or axonal loss within the pathways, which could give rise to the abnormal somatosensory cortical processing observed in CP (Papadelis et al. 2014; Pihko et al. 2014; Nurmi et al. 2021; Illman et al. 2023).

The efficient functioning of the somatosensory system is essential for the smooth execution of movements, i.e. for motor control. Recent studies have therefore aimed to clarify how the structure of the thalamocortical pathways and sensorimotor performance are associated in CP. Indeed, more pronounced white matter abnormalities of the thalamocortical pathways have been associated with a wide variety of measures quantifying upper-limb sensorimotor performance in spastic CP (Hoon et al. 2009; Pannek et al. 2014; Tsao et al. 2015; Kuczynski et al. 2017; Papadelis et al. 2018; Mailleux et al. 2020b). Additionally, it has been suggested that hemispheric symmetry of the thalamocortical tracts could be important for better gross motor performance in CP (Thomas et al. 2005; Rose et al. 2011; Tsao et al. 2015; Reid et al. 2017). Research addressing the interplay between thalamocortical connections and lower-limb function in CP has, however, been scarce (Mailleux et al. 2020a).

Methodologically, most studies in the field have been limited by the lack of detailed tractography when quantifying the thalamocortical projections. Typical analyses include all streamlines between the thalamus and a broad cortical area such as the postcentral gyrus, despite the well-known somatotopy of the SMI cortices (Penfield and Jasper 1954). In recent years there have been advances in pinpointing the tracts connecting to functionally relevant upper-limb cortical areas in CP using functional imaging (Reid et al. 2016; Papadelis et al. 2018). Moreover, recent studies have suggested that more detailed structural differences can be found when the dMRI metrics are not averaged over the whole course of the pathways (Jiang et al. 2019; Jaatela et al. 2023).

Here, the primary aim was to use novel tractography methods to evaluate how thalamocortical pathways are impaired in spastic CP. Following the tractography pipeline developed by our group (Jaatela et al. 2022), we constructed separate thalamocortical tracts specific for the representational areas of each of the four extremities. First, the limb-specific cortical seeds were selected manually based on anatomical landmarks of the primary somatosensory cortex, and then based on fMRI activations induced by passive-movement-evoked proprioceptive stimulation of each limb separately in a subset of our participants. The functional relevance of the tractography observations was further assessed by investigating the respective relationships to behavioral sensorimotor performance of the upper (fine and gross motor hand skill) and lower limbs (static and dynamic stability). We hypothesized that the limb-specific tracts would be differently impaired in hemiplegic and diplegic CP, following the topographical differences in the sensorimotor symptoms between the two CP subtypes. In addition, we expected that the magnitude of these white matter changes and the severity of sensorimotor impairments would be associated.

## Materials and methods

### Participants

#### Patients

In total, 25 children and adolescents with spastic CP participated in the dMRI study (for the demographics summary see Supplementary Table S1). A total of 15 had a confirmed diagnosis of hemiplegic CP (HP: *N* = 15, 11 females, age: 13.7 ± 2.2 years, range: 10.9–18.1 years) and 10 diplegic CP (DP: *N* = 10, 5 females, age: 13.4 ± 2.0 years, range 10.8–16.7 years). All were able to walk independently (GMFCS level I–II). Except for one HP and one DP participant with Full-Scale IQ under 70, all were cognitively within normal variation (Wechsler Adult Intelligence Scale/Wechsler Intelligence Scale for Children; Full-Scale IQ score: 95.3 ± 17.8, range: 43–117; three participants missing a test score). They had no known cognitive or co-operative deficiencies, hearing deficit, visual deficit other than refractive error, or condition (other than CP) or medication known to affect sensorimotor performance. The hemiplegic group included six right-handed and nine left-handed participants when the dominant hand was defined as the less-affected hand. The diplegic group included six right-handed and four left-handed participants when assessed with the Edinburgh Handedness Inventory test (mean score: 19.8, range: −100 to 100; Oldfield 1971).

#### Controls

The same participants described previously by Jaatela et al. (2022) constituted the control group. In short, 19 typically developed participants (TD: *N* = 19, 14 females, age: 14.2 ± 2.5 years, range: 10.5–17.7 years) were all right-handed (Edinburgh Handedness Inventory test, mean score: 82.7, range: 43–100). All controls were cognitively within normal variation (Wechsler Adult Intelligence Scale/Wechsler Intelligence Scale for Children; Full-Scale IQ score: 108.0 ± 14.9, range: 77–132; three participants missing a test score) and had no known neurological deficits or medication.

The study was approved by the Helsinki University Hospital ethics committee (HUS/2318/2016) and was in accordance with the recommendations of the Declaration of Helsinki. Informed written consent was obtained before the experiment from all participants and their guardians.

### Sensorimotor performance

The sensorimotor testing of the upper and lower extremities was conducted on a separate day either before or after the MRI session. Sensorimotor performance of the upper limbs was assessed with Box and block test (developed by Hyres and Buhler 1957; Mathiowetz et al. 1985a) and Nine-Hole Peg test (Mathiowetz et al. 1985b). The Box and block test evaluates unilateral gross-manual dexterity by counting the number of blocks moved from one box to another in 60 s. The Nine-Hole Peg test quantifies unilateral fine-manual dexterity by measuring the time it takes to place and remove nine pegs into nine holes.

Sensorimotor performance of the lower limbs was assessed using static and dynamic stability tests. Static stability was quantified as the postural sway (center-of-force velocity, mm/s) during standing with eyes open or closed, feet apart or together. Dynamic stability was measured as body acceleration (refined-compound-multiscale entropy; Ihlen et al. 2016) of inertial measurement unit during normal gait, motor dual task, and cognitive dual task (for more detailed information, see Jaatela et al. 2023; Piitulainen et al. 2021).

To obtain a single latent estimate of the overall upper and lower limb performances and increase the statistical power, we constructed sum variables from the individual tests separately for the upper and lower limbs. In order to ensure a balanced impact of individual test scores on the overall sum variables, the single test scores were first normalized across all participants using min–max normalization (0 = worst performance in the sample, 1 = best performance in the sample, every other value between 0 and 1). If a participant was missing less than half of the test scores, the sum variable could be estimated. However, to avoid undesirable emphasis of the non-missing scores in such cases, the missing values were replaced by the average normalized value of the correct group (TD, HP, or DP). The normalized scores were then averaged for each participant to construct single sum variables for *hand skill* (including the measures for both dominant and non-dominant hand) and *stability*. The internal consistency of the sum variables was estimated with Cronbach’s alphas and 0.8 was considered as the limit for good internal consistency (Cronbach 1951; Nunnally and Bernstein 1994).

### The dMRI data acquisition and processing

The details of the used dMRI protocol and analysis pipeline have been previously reported by Jaatela et al. (2022). To summarize, the imaging was performed with a 3 T scanner and 32-channel head coil at Aalto NeuroImaging. The dMRI was acquired in 64 gradient directions with *b* = 1,000 s/mm^2^ and additional eight images with *b* = 0 s/mm^2^ (echo-planar imaging, voxel size = 2.5 mm^3^; field-of-view = 240 × 240 mm; reconstructed matrix = 96 × 96; slices = 70; TR/TE = 8.3 s/81 ms; flip angle = 90°). Images with *b* = 0 were gathered in both posterior–anterior and anterior–posterior phase encoding direction to create an estimate of the susceptibility-induced off-resonance field.

First, eddy current and motion corrections were applied with FMRIB’s Software Library tools eddy and topup (FSL version 6.0; Andersson et al. 2003; Smith et al. 2004; Jenkinson et al. 2012). Then, ExploreDTI software (version 4.8.6; Leemans et al. 2009) was used to co-register the diffusion-weighted images to the T1-image and interpolate dMRI data to 1 mm^3^ voxel size. The co-registration also allowed us to use anatomical labels for tractography. ExploreDTI software was further utilized to estimate voxel-wise diffusion tensors with the REKINDLE algorithm (Tax et al. 2015). The fiber orientation distributions (FOD) were estimated with 8th order spherical harmonics compartment model approach (Tran and Shi 2015), which can separate the intra- and extra-axonal components of the signal and address complex structures such as crossing fibers.

### Creating the regions-of-interest for tractography

For the construction of limb-specific thalamocortical tracts, a set of regions-of-interest (ROIs) was defined in the individual anatomy of each participant following the pipelines introduced by Jaatela et al. (2022). The cortical ROIs for the upper and lower limbs were first marked manually and then, in a subset of participants, using an fMRI-based pipeline. The Freesurfer image analysis suite (http://surfer.nmr.mgh.harvard.edu/; Dale et al. 1999; Fischl et al. 2002; Segonne et al. 2007; Fischl 2012) was used for the cortical reconstruction and volumetric segmentation of the T1-weighted images. The cortical and white matter surface reconstructions were visually checked and thalamic segmentations (Fischl et al. 2002) were further verified on the color-coded FA map.

#### Manual-ROIs

First, we marked the cortical ROIs based purely on the anatomical landmarks of the hand and foot areas of the contralateral SMI cortex (Jaatela et al. 2022). These single-voxel (1 mm^3^) manual-ROIs were carefully placed on the post-central sulcus white matter surface to ensure standardized tractography (Guye et al. 2003; Smits et al. 2007; Oechslin et al. 2018). Selecting the ROIs manually based on known cortical anatomy is valid when no functional imaging data is available, but the selection can be challenging in the presence of cortical lesions or malformations. The placement of manual ROIs was performed by author JJ.

#### fMRI-ROIs

For comparison, fMRI-seeded tractography was performed for a subset of participants. The fMRI pipeline with proprioceptive stimulation has been previously described by Jaatela et al. (2022) and in more detail by Nurmi et al. (2021). In short, the stimulation blocks of the fMRI scans [echo-planar imaging, voxel size = 3 mm^3^; field-of-view = 192 × 192 mm; reconstructed matrix = 64 × 64; slices = 44; TR/TE = 2.5 s/30 ms; flip angle = 90°] consisted of continuous passive movements of each limb separately: (i) right index finger, (ii) left index finger, (iii) right ankle, and (iv) left ankle. Preprocessing contained slice-time-correction, motion-correction, co-registration to T1, correction of respiration and pulsation-induced artifacts (Drifter tool, Särkkä et al. 2012), smoothing and temporal high-pass filtering (SPM12 software: Wellcome Department of Imaging Neuroscience, University College London, UK; Matlab R2016b: Mathworks, Natick, Massachusetts, United States). The threshold of the general linear model contrast images was adjusted to show visible activation in the contralateral SMI cortex for each stimulus (Marsbar toolbox: MARSeille Boîte À Région d’Intérêt; Marseille, France; version 0.44). For each limb, the activated area was limited to an 8 mm sphere around the local maximum, and from this area, a center-of-mass was selected as the primary activation location. The center-of-mass was finally projected manually on the individual white matter surface resulting in a single-voxel fMRI–ROI.

### Probabilistic tractography

Similarly to Jaatela et al. (2022), tractography was performed with parallel transport tractography algorithm (Aydogan and Shi 2020) in Trekker (https://dmritrekker.github.io/) with the following parameters: probeLength = 0.2 mm, minRadiusOfCurvature = 0.4 mm, minFODamp = 0.01 and dataSupportExponent = 0.25, other parameters were set to default. At first, 1 million thalamocortical tracts were generated using random voxels within the Freesurfer-derived left or right thalamus as starting points for left or right hemisphere tracts. Fibers jumping across any sulcus or the brain’s midline were removed. Then, the streamlines were filtered to reach the limb-specific manual-ROIs or fMRI–ROIs. To address the possible registration errors, the original 1 mm^3^ cortical ROIs were dilated using a spherical kernel of 3 mm.

After filtering the limb-specific tracts, spurious streamlines were excluded with coherence measure thresholding (Meesters et al. 2016) using Dipy (Garyfallidis et al. 2014) and visually verified. Tract masks were obtained by marking all non-zero voxels in the MRtrix3-computed (Tournier et al. 2019) tract density images (Calamante et al. 2010) as one. To evaluate the microstructural properties of the tracts, we mapped the FA, MD, and apparent fiber density (AFD; Raffelt et al. 2012) of the tract masks. These metrics were averaged over all streamlines to compare the average values of limb-specific tracts across the groups. Additionally, the hemispheric asymmetry of these dMRI metrics was evaluated for upper and lower limb thalamocortical tracts using Asymmetry Index (AI): [(Value of the non-dominant tract—Value of the dominant tract)/((Value of the non-dominant tract + Value of the dominant tract)/2)] × 100. Finally, along-tract analysis was performed for each limb-specific tract using the median values of all streamlines belonging to the bundle. For example, in the first tract point (i.e. on the thalamus surface) we took the first value of all streamlines and selected the median. Moving away from the thalamus toward the cortex, the same was repeated until the distance was reached by less than five streamlines.

### Statistical analysis

Statistical analyses were performed with R statistical software (version 4.2.1; R Development Core Team, 2022). The Kruskal Wallis H-test (Kruskal and Wallis 1952) was applied to check for statistically significant (*P* < 0.05) differences between the groups. If a difference was found, the Conover post-hoc test with FDR correction (Benjamini and Hochberg 1995; Conover 1999) was used to determine pair-wise differences. For along-tract analysis, we used the H-test without post-hoc tests.

For correlation analysis within each group, we applied the two-tailed Pearson correlation corrected for age (Kim 2015). The correlations were studied (i) between upper-limb manual-seeded tract FA/MD/AFD values and hand skill and (ii) between lower-limb manual-seeded tract FA/MD/AFD values and stability. Absolute values of asymmetry indices were used for correlation analysis to improve the interpretation of the results.

## Results

### Hand skill and stability were impaired in CP participants

**Fig. 1:**
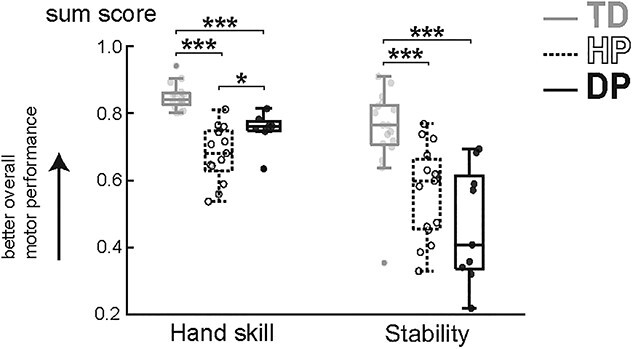
Sum variables for the upper and lower limb performance, i.e. hand skill and stability. Larger values indicate better overall performance. ^*^  *P* < 0.05, ^*^^*^  *P* < 0.01, ^*^^*^^*^  *P* < 0.001.

The sensorimotor sum variables for each group are presented in Fig. 1 and the results from individual tests are listed in Supplementary Table S2**.** The final hand skill data included 16 controls and 20 patients (13 HP, 7 DP) and stability data 17 controls and 24 patients (15 HP, 9 DP). Some participants did not participate in sensorimotor testing due to their schedule challenges (2 TD, 1 DP), and some participated only in stability testing (1 TD, 2 HP, and 2 DP). Imputation of test values was used for two hemiplegic and one diplegic CP participants for hand skill, and for one control and two diplegic participants for stability. The internal consistency of the hand skill and stability performance sum variables were good (Cronbach alphas: 0.84 for hand skill and 0.91 for stability).

**Table 1:** The number of participants with limb-specific thalamocortical tracts successfully extracted.

	Hemiplegic cerebral palsy		Diplegic cerebral palsy		Typically developed	
	Manual	fMRI	Manual	fMRI	Manual	fMRI
Non-dominant upper-limb tract	9	9	10	5	19	19
Dominant upper-limb tract	14	9	10	5	18	17
➔ AI for upper-limb tracts	9	8	10	5	18	17
Non-dominant lower-limb tract	13	9	9	5	19	17
Dominant lower-limb tract	14	9	9	5	19	19
➔ AI for lower-limb tracts	13	8	8	5	19	17

Tracts were obtained either using manual-ROIs or fMRI-ROIs. Asymmetry indices (AI) were calculated separately for upper and lower limb tracts.

fMRI = functional magnetic resonance imaging; AI = asymmetry index; ROIs = regions-of-interest.

The lowest hand skill values (i.e. worst gross and fine manual performance) were observed in hemiplegic CP (*P* < 0.001 compared to TD, *P* = 0.045 compared to DP). Also, the diplegic participants showed decreased hand skill values compared to controls (*P* < 0.001). The hand skill sum variable was calculated by pooling both dominant and non-dominant hand results together. Stability values (i.e. dynamic gait and static standing performance) were decreased in both hemiplegic and diplegic participants when compared to controls (*P* < 0.001 for both). Participants with diplegic CP appeared to show the lowest stability values although no statistical significance was seen between the two CP groups.

### Both upper and lower limb thalamocortical tracts were affected in CP

The dMRI data of all 44 participants was of satisfactory quality when assessed visually. Due to deviant anatomy, i.e. malformation or lesion over the SMI cortex (for an example, see Fig. 4A), we were unable to place the manual-ROIs for some hemiplegic participants (all manual-ROIs for 1 HP and non-dominant upper-limb ROIs for 4 HP). We were unable to obtain the fMRI-ROISs for some patients for the following reasons: inability to perform the finger stimulation due to spasticity (1 HP), interrupted measurement due to discomfort in the scanner (1 HP) and excessive head movement during the fMRI scan (2 HP, 5 DP). Additionally, some fMRI-ROIs were excluded due to clearly deviant spatial locations combined with unsatisfactory data quality (3 TD, 3 HP). Finally, five fMRI-seeded (1 TD, 4 HP) and three manual-seeded tracts (1 TD, 2 DP) were excluded due to low (<5) streamline counts. Table 1 lists the final numbers of analyzed tracts obtained with either manual-ROIs or fMRI–ROIs. It is noteworthy that all limb-specific tracts did not include identical participants. Fig. 4A shows examples of the constructed tracts.

**Fig. 2:**
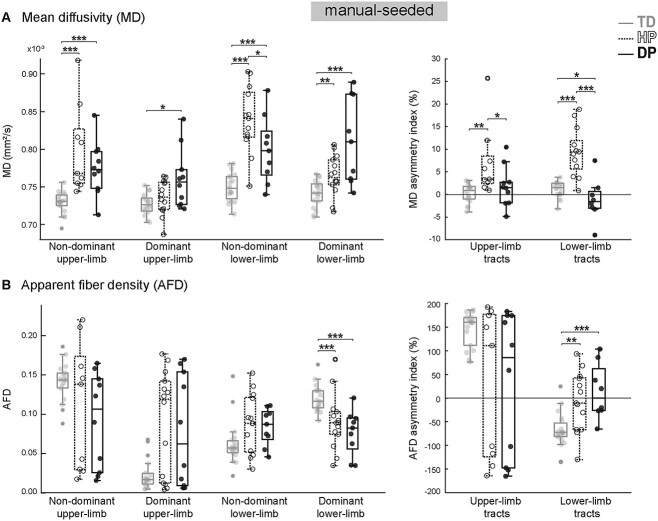
Manual-seeded thalamocortical tracts. A) MD values of dominant and non-dominant upper and lower limb thalamocortical tracts and the corresponding asymmetry indices. B) Tract-specific AFD values and corresponding asymmetry indices. ^*^  *P* < 0.05, ^*^^*^  *P* < 0.01, ^*^^*^^*^  *P* < 0.001.

**Fig. 3:**
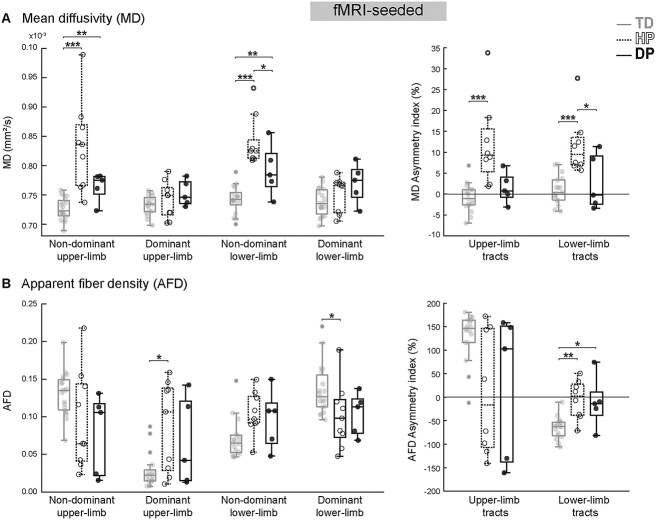
FMRI-seeded thalamocortical tracts. A) MD values of dominant and non-dominant upper and lower limb thalamocortical tracts and the corresponding asymmetry indices. B) Tract-specific AFD values and corresponding asymmetry indices. ^*^  *P* < 0.05, ^*^^*^  *P* < 0.01, ^*^^*^^*^  *P* < 0.001.

#### Manual-seeded tracts

Figure 2 illustrates the MD and AFD values averaged over the limb-specific tract bundles obtained with manual seeding. MD, describing the total diffusion magnitude, was significantly increased in both CP groups. As hypothesized, the hemiplegic participants seemed to be affected more in the non-dominant hemisphere (*P* < 0.001 for non-dom. Tracts; *P* = 0.005 for dom. Lower-limb tract), compared to the more bilateral involvement in the diplegic group (*P* < 0.001 for non-dom. Tracts; *P* = 0.017 for dom. Upper-limb tract; *P* < 0.001 for dom. Lower-limb tract). Moreover, the hemiplegic participants showed higher MD in the non-dominant lower-limb tract compared to those with diplegia (*P* = 0.031). The AFD, describing the fiber density, was decreased significantly in the dominant lower-limb tract in patients compared to controls (*P* < 0.001 for both HP and DP). The anisotropy value, FA, did not differ between the groups (*P* = 0.07–0.88).

**Fig. 4:**
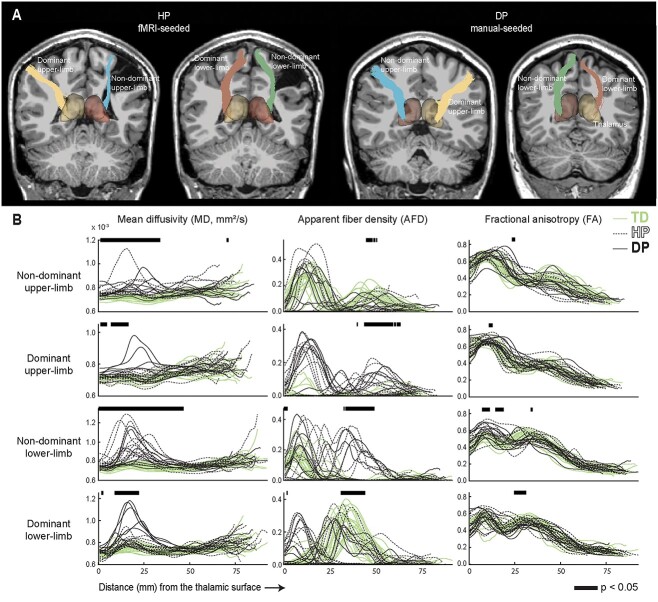
Along-tract analysis. A) Limb-specific thalamocortical tracts of representative CP participants obtained with fMRI-seeding or manual-seeding. (B) MD, AFD and FA values along the manual-seeded thalamocortical tracts. Black lines on top of each graph indicate the positions where the three groups differ significantly (*P* < 0.05, H-test) from each other. For illustration, we used smoothing with a 10-point moving average. Position 0 is located on the thalamus surface.

Following the tract-specific results, the hemispheric asymmetry of MD and AFD, but not FA, showed intergroup differences. Children with hemiplegic CP had increased MD asymmetry (indicating higher MD in the non-dom. tracts) compared to both controls (*P* = 0.002 and *P* < 0.001 for upper and lower limb tracts, respectively) and diplegic CP (*P* = 0.027 and *P* < 0.001 for upper and lower limb tracts, respectively). The asymmetry of lower-limb tracts’ MD was in contrast decreased in diplegic participants compared to controls (*P* = 0.030).

The AFD asymmetry of the lower-limb tracts was increased in patients compared to controls (*P* = 0.001 and *P* < 0.001 for HP and DP, respectively). It is noteworthy, that the lower-limb AFD asymmetry in the control group was negative, indicating higher AFD values in the dominant hemisphere, whereas the patient groups were closer to zero, i.e. to hemispheric symmetricity. Interestingly, the AFD asymmetry of the upper-limb tracts in controls was in contrast positive.

#### fMRI-seeded tracts

The MD and AFD values of the fMRI-seeded tracts, presented in Fig. 3, resembled those obtained with manual seeding. Again, no statistically significant differences were detected in the tract-specific FA values or in the FA asymmetry (*P* = 0.20–0.90).

In the non-dominant hemisphere, MD values were increased in both hemiplegic (*P* < 0.001 for upper and lower limb tracts compared to TD; *P* = 0.030 for lower-limb tract compared to DP) and diplegic patient groups (*P* = 0.002 and *P* = 0.007 for upper and lower limb tracts, respectively, compared to TD). The diplegic participants appeared to have increased MD values also on the dominant side, although no statistical significance was detected. The AFD values of the dominant lower-limb tract were again lower in participants with hemiplegic CP compared to controls (*P* = 0.044), whereas for the dominant upper-limb tract, the AFD was higher (*P* = 0.033). Although the AFD values of the diplegic group resembled those obtained with manual seeding, the differences were not statistically significant.

Following the manual-seeded tract results, children with hemiplegic CP showed increased MD asymmetry compared to both controls (*P* < 0.001 for upper and lower limb tracts) and diplegic CP (*P* = 0.022 for lower-limb tracts). Here, however, no statistically significant difference in MD asymmetry was found between diplegic participants and controls. The AFD asymmetry for lower-limb tracts was again increased in both CP groups compared to controls (*P* = 0.004 and *P* = 0.015 for HP and DP, respectively).

Finally, we noted that the AFD values had high variability in patients’ upper-limb tracts. In both manual and fMRI-seeded tracts, some patients had clearly negative and some clearly positive AFD asymmetry. With closer inspection, we observed that all CP participants with negative asymmetry values (i.e. AFD higher on the dominant upper-limb tract) had a dominant left hand, whereas participants with positive values had a dominant right hand. Furthermore, the opposite seemed to be true for lower-limb tracts: positive AFD asymmetry was associated with left-hand dominancy. Based on these observations, it seems that the AFD asymmetry measure might be driven by left–right orientation, rather than limb dominancy. Moreover, this asymmetry seems to be different for upper and lower limb tracts.

### Diffusion properties varied along the thalamocortical tracts

Figure 4B shows the along-tract analysis of the dMRI metrics revealing distinct trajectories for patient and control groups. Statistically significant (*P* < 0.05) intergroup differences on the manual-seeded tracts were observed in distinct positions for the three dMRI metrics. These trajectories appeared similar in the fMRI-seeded tracts with some variation in the exact *P*-values (see Supplementary Fig. S1).

As seen from Fig. 4B, thalamocortical tract MD values differed between the three groups especially in the non-dominant side, although differences were seen also in the dominant hemisphere tracts. It can be observed that CP groups had increased MD values closer to the thalamus, and the values were more scattered in all groups when moving closer to the cortex. The intergroup differences in the AFD values were located in the tract sections further away from the thalamus. The FA values seemed similar across groups, with only short individual sections differing between the groups.

Figure 4B further shows that the controls had distinct limb-specific AFD trajectories. In the non-dominant upper-limb tract, the AFD values had two distinguishable peaks (first peak ~10 mm away from the thalamic surface, second ~45 mm away). In the non-dominant lower-limb tract, the first peak looked somewhat similar whereas the second peak was not distinguishable. The dominant lower-limb tract showed only one peak (~35 mm away from the thalamus) and the dominant upper-limb tract did not show any clear peaks in the AFD values. In both CP groups, there was clearly more variability in the peak pattern although some patients also followed the control group trajectories.

### Associations between thalamocortical tract structure and sensorimotor performance

Table 2 lists the age-corrected correlations between thalamocortical tract properties and sensorimotor performance (i.e. between upper-limb tracts and hand skill, and between lower-limb tracts and stability) and correlations with *P* < 0.05 are illustrated in Supplementary Fig. S2. After correcting for multiple comparisons (Bonferroni, *P* < 0.05/9 ≈ 0.006), none of the correlations were significant.

Nevertheless, negative correlations seemed to exist in the control groups between the FA asymmetry and the sensorimotor performance of both the upper (*r* = −0.60, *P* = 0.019) and lower extremities (*r* = −0.50, *P* = 0.046). Negative values indicate that the hemispheric symmetricity of the upper-limb thalamocortical tracts was associated with better hand skill performance and likewise for lower-limb tracts and stability performance. In the diplegic groups, the MD asymmetry for upper-limb tracts seemed to correlate negatively with the hand skill (*r* = −0.82, *P* = 0.048), indicating a similar relationship between hemispheric symmetricity and performance. In contrast, the upper-limb tracts’ AFD asymmetry was observed to correlate positively with hand skill in diplegic participants (*r* = 0.92, *P* = 0.010), indicating an association between less symmetric AFD values and better hand skill performance. As the control group showed asymmetric AFD both in upper and lower limb tracts, it can be expected that AFD asymmetricity was associated with more typical motor performance.

The hemiplegic group showed no significant correlations in the asymmetry measures, but the dominant upper-limb tract appeared to be negatively correlated with the hand skill (*r* = −0.60, *P* = 0.041). A negative correlation was found also between non-dominant lower-limb tract AFD and stability in the diplegic group (*r* = −0.71, *P* = 0.031). These relationships indicate an association between lower AFD values and better sensorimotor performance. It is noteworthy that in controls, the AFD values varied between the limb-specific tracts, but for dominant upper-limb and non-dominant lower-limb tracts the AFD values were low in controls.

**Table 2:** Age-corrected Pearson correlations between manual-seeded thalamocortical tracts and sensorimotor performance (sum variables: hand skill and stability).

		Hemiplegic cerebral palsy	Diplegic cerebral palsy	Typically developed
**Corralations with hand skill** ^a^				
	Non-dominant upper-limb tract	−0.05	−0.33	−0.01
FA	Dominant upper-limb tract	0.10	−0.66	−0.19
	AI for upper-limb tracts	0.23	0.13	−0.60^*^
	Non-dominant upper-limb tract	−0.07	−0.17	−0.15
MD	Dominant upper-limb tract	0.44	0.22	−0.41
	AI for upper-limb tracts	−0.06	−0.82^*^	0.17
	Non-dominant upper-limb tract	0.56	−0.51	−0.08
AFD	Dominant upper-limb tract	−0.60^*^	−0.10	−0.40
	AI for upper-limb tracts	−0.03	0.92^*^^*^	0.09
**Correlations with stability**				
	Non-dominant lower-limb tract	0.13	−0.57	0.12
FA	Dominant lower-limb tract	−0.37	−0.32	0.19
	AI for lower-limb tracts	0.42	−0.15	−0.50^*^
	Non-dominant lower-limb tract	0.38	0.20	0.02
MD	Dominant lower-limb tract	0.04	0.39	−0.24
	AI for lower-limb tracts	0.32	0.02	−0.45
	Non-dominant lower-limb tract	0.24	−0.71^*^	0.13
AFD	Dominant lower-limb tract	−0.18	−0.02	0.31
	AI for lower-limb tracts	−0.19	0.37	0.01

^a^Unimanual upper-limb tests were pooled together in the sum variable.

FA = fractional anisotropy; MD = mean diffusivity; AFD = apparent fiber density; AI = asymmetry index.

^*^  *P* < 0.05; ^*^^*^  *P* < 0.01; After correcting for multiple comparisons no correlations were significant.

## Discussion

This study set out to evaluate how the limb-specific thalamocortical white matter pathways are affected in hemiplegic and diplegic CP. We showed that MD was significantly increased in CP compared to controls, whereas no significant differences were detected in FA values. As hypothesized, the thalamocortical projections were bilaterally affected in diplegic CP, and more unilaterally affected in hemiplegic CP. The hemispheric asymmetricity of thalamocortical AFD values was observed in controls but not in children with CP. The results of anatomy-based manual-seeded projections were confirmed with fMRI-guided tractography. Finally, we observed correlations between the thalamocortical tract properties of the upper and lower limbs, and hand skill and lower-limb stability performances, respectively.

### Mean diffusivity of the thalamocortical tracts was increased in CP

The MD was increased in thalamocortical tracts both in hemiplegic and in diplegic participants, supporting earlier findings in CP (Mailleux et al. 2020a). Higher MD indicates less restricted diffusivity within the studied voxel and is thought to be related to lower tissue complexity or neuronal density (Curran et al. 2016). As hypothesized, the dominant and non-dominant hemispheres were affected differently in the two CP groups. Diplegic participants showed elevated MD values bilaterally, whereas hemiplegic participants had increased values prominently on their non-dominant side. Furthermore, the MD asymmetry was increased in the hemiplegic group compared to both diplegic and control groups. Similar results have been previously reported by Papadelis et al. (2014) who showed that children with hemiplegic CP had a more pronounced increase of the thalamocortical diffusivity values in their more affected hemisphere compared to the less affected hemisphere than those with diplegic CP. More asymmetric diffusivity in hemiplegic than diplegic CP has also been reported in the efferent corticospinal tract (Cho et al. 2013). Our results reflect previous studies both in hemiplegia, reporting increased diffusivity values especially in the affected hemisphere (Lennartsson et al. 2015; Tsao et al. 2015; Kuczynski et al. 2017; Papadelis et al. 2018, 2019), and in diplegia, showing minor asymmetry between the hemispheres (Rha et al. 2012).

While in diplegic CP the prominent clinical dysfunction is in the lower extremities, it was interesting that the increased MD values were observed also in the thalamocortical tracts projecting to the upper-limb representation areas. However, despite the emphasis on lower-limb functioning, also hand performance can be diminished in diplegic CP (Rosenbaum et al. 2007). This was observed in our hand skill measure (pooled over both hands) where the diplegic group showed worse performance than their typically developed peers. Furthermore, in a partly overlapping sample, Piitulainen et al. (2020) showed that cortical processing following proprioceptive stimulation of the finger was altered in diplegic CP, whereas no differences were found between hemiplegic and typically developed participants. Additional explanation for the similar MD values in upper and lower limb tracts is that the elevated MD values were observed closer to the thalamus surface, where both upper and lower limb tracts follow the same white matter bundle and thus share more identical dMRI voxels. To our knowledge, this is the first time that thalamocortical tracts for upper and lower limbs were studied separately in the same CP participants. Similar results have been, however, presented earlier by Chang et al. (2012) in the corticospinal tract, showing that children with diplegic CP had increased diffusivity compared to controls in their upper as well as lower limb tracts. Moreover, diplegic CP has been associated with widespread alterations of the white matter network (Lee et al. 2017).

While the diffusivity measure is considered to indicate changes in tissue complexity or neuronal density (Curran et al. 2016), the elevated MD values in CP may lead to altered thalamocortical neuronal activity and contribute to the abnormal somatosensory processing. In support of this reasoning, Papadelis et al. (2018) showed that the higher diffusivity of the non-dominant finger thalamocortical tract correlated with the stronger MEG response amplitude following a tactile stimulus of the finger in hemiplegic CP. Our previous fMRI results have also demonstrated that the blood-oxygen-level-dependent responses to proprioceptive stimulation of the non-dominant finger were elevated in adolescents with CP (partly overlapping with the current participants; Nurmi et al. 2021). The stronger functional cortical responses can thus indicate inefficient neural processing caused by the impaired thalamocortical projections (Papadelis et al. 2018; Nurmi et al. 2021), although, future work is required to clarify the relationship between specific white matter structures and cortical processing.

One alternative explanation for the increased MD could be that diminished connectivity between other brain areas decreases the number of crossing fibers within the studied voxels and increases the measured diffusivity (Vos et al. 2012). In addition, different maturational levels or trajectories between the groups may affect the measured diffusivity as it is known to decrease through adolescence (Snook et al. 2005; Blakemore 2012) and the developmental changes of the thalamocortical pathways continue to adulthood (Alkonyi et al. 2011). Our control and patient groups were age-matched, but the influence of the maturational aspects is still possible. Finally, the partial volume effect in the proximity of enlarged ventricles or lesions cannot be excluded as a confounding factor. Indeed, some of our CP participants showed high MD values in the along tract analysis close to the lesion locations. MD is more prone than FA to the partial volume effect, and narrow pathways increase the risk while the “edge” of the fiber bundle weighs more in averaging (Curran et al. 2016). However, not all increased MD values were located in the proximity of an area filled with cerebrospinal fluid, and in order to minimize this effect, we used the median values in the along-tract analysis.

Unlike some previous studies (Papadelis et al. 2014; Tsao et al. 2015; Kuczynski et al. 2017), we did not observe reduced FA values of the thalamocortical tracts in the whole-tract-level. However, many studies have also reported that the FA values of thalamocortical projections seem not to differ between children with CP and controls (Pannek et al. 2014; Lennartsson et al. 2015; Papadelis et al. 2018, 2019). It has been suggested that the number and volume of the thalamocortical fibers (reflected by increased MD) might be decreased in CP, but the integrity of the remaining fibers is preserved (reflected by unhanged FA) (Rha et al. 2012). However, we did observe some group-wise differences in the along-tract analysis of FA values between the studied groups. Subtle FA changes cannot thus be ruled out, especially as the sample size was limited in our study, as well as in most of the previous CP studies. It is well established that the etiology in CP is very heterogenous thus also explaining inconsistencies between studies.

### Apparent fiber density of the thalamocortical tracts appeared less lateralized in CP

The AFD has been previously shown to be strongly lateralized in the thalamocortical tracts of typically developed adolescents (Jaatela et al. 2022) but the same was not observed in the participants with CP. For the upper-limb thalamocortical tracts, the AFD variance within patients was high and there were no statistically significant differences in the asymmetry values compared to controls. For the lower-limb tracts, both hemiplegic and diplegic CP participants showed more hemispheric symmetry compared to controls.

Although thalamocortical projections are reported to show lateralization in typical development (Alkonyi et al. 2011; Fling et al. 2014), it is surprising that the AFD asymmetricity is the opposite for upper and lower limb tracts (Jaatela et al. 2022). The research on the hemispheric specialization of the thalamocortical tracts is limited, and it is unknown whether it is dependent on handedness. The corticospinal tracts appear to be left-lateralized in both left and right-handed individuals, although some studies show that in certain areas, i.e. in the motor hand area, the laterality can be weaker or even reserved for left-handers (for review, see Budisavljevic et al. 2021). Our control group was solely right-handed, whereas approximately half of the children with CP were left-handed (i.e. right hand was more affected). Thus, the intergroup differences observed in the AFD measures might be driven by the hemispheric specialization that is independent of hand dominancy. It is also possible that the developmental trajectory of the hemispheric specialization is altered in response to injury in children with CP (Ferre et al. 2020). These questions are, however, out of the scope of the current study, and future work including left-handed healthy individuals is needed to clarify the lateralization of limb-specific white matter pathways.

Despite the possible influence of hemispheric lateralization, we showed distinct AFD trajectories for limb-specific tracts that seemed to differ between CP groups and controls. Although some individuals with CP seemed to follow the control group trajectories more closely, some had visibly deviant AFD values along the thalamocortical pathways. While AFD is sensitive to changes in both the diffusion characteristics and white matter volume (Raffelt et al. 2012), these individual trajectories can reveal structural changes in the thalamocortical tracts that are not visible with traditional dMRI metrics such as FA and MD.

### Benefit of fMRI-seeded tractography in examining patients with abnormal cortical anatomy

In the presence of lesions and other malformations that are common in the CP brain, functional imaging could be beneficial to pinpoint the cortical areas that are associated with the white matter connections under study (Reid et al. 2016; Papadelis et al. 2018). Here, we showed that the manual-seeded and fMRI-seeded thalamocortical tracts yielded similar results in CP. Although we were unable to compare the two seeding approaches in identical participants due to the limited sample size, our results provide support to the premise that functional seeding might serve as an alternative method in patients with abnormal cortical anatomy when the typical landmarks such as the hand-knob are missing. However, more research is required to confirm the reliability of thalamocortical tractography based on proprioceptive fMRI activation.

To the best of our knowledge, this is the first paper reporting and comparing tractography results using both manual (based purely on anatomy) and functional (based on proprioceptive fMRI responses) seeding approaches in adolescents with CP. We have previously shown that the fMRI and manual approaches provide similar diffusion metrics in the limb-specific thalamocortical tracts in typically developed individuals (Jaatela et al. 2022), and thus it was expected that these two seeding methods would be comparable. A drawback of the method is that the fMRI protocol often elicits several activation clusters, thus introducing a partially subjective element to the pipeline. Furthermore, a disadvantage of this study was that the participant with hemiplegic CP with large cortical lesions or malformations were the ones most likely excluded from the manual-seeded analysis, although they are the ones likely to benefit the most from functional seeding. However, at the group level, the thalamocortical tract properties in the hemiplegic CP appeared strikingly similar. For future studies, it would be beneficial to evaluate the two seeding methods also on the individual level and possibly concentrate on CP participants with an abnormal cortical organization.

Our objective with the fMRI protocol was to identify the specific cortical areas that receive input from the pathways transmitting sensory information from the proprioceptors. Although thalamus is densely connected to other SMI areas as well, we decided to target specifically the proprioceptive afference, given the importance of proprioception in manual dexterity and stability. Previous research by Van de Winckel et al. (2013) demonstrated that in children with hemiplegic CP, the passive movement of the index finger activated the contralateral SMI as well as SI cortical regions, which was not observed with voluntary movements. Nevertheless, there is a scarcity of studies utilizing neuroimaging techniques to explore proprioceptive afference, and as a result, the optimal approaches for identifying the regions responsible for proprioceptive processing remain unclear.

### Limb-specific tractography was correlated with sensorimotor performance

To evaluate the functional relevance of the thalamocortical white matter structure, we studied the association between upper-limb tracts and hand skill, and between lower-limb tracts and stability. We found moderate correlations in all groups, although none of which survived correction for multiple comparisons. However, some conclusions can be drawn as the correlation directions were consistent and expected: when compared against the typically developed group, the more altered the dMRI metric was, the worse the sensorimotor performance.

Our findings were in accordance with previous studies indicating that increased asymmetry of the thalamocortical tracts is connected to worse sensorimotor performance. It could be that the association is driven by the structure–function relationship of the non-dominant (more affected) side as displayed in multiple studies in participants with hemiplegic CP (Hoon et al. 2009; Rose et al. 2011; Tsao et al. 2015; Kuczynski et al. 2017; Papadelis et al. 2018). The integrity of the more affected thalamocortical tract relates to better sensorimotor performance and results simultaneously in smaller hemispheric asymmetry. This view is supported by the findings in healthy adults by Reid et al. (2017) who showed that unimanual training of the non-dominant hand resulted in increased FA in the contralateral corticospinal tract, and thus lower hemispheric asymmetry. However, it is also possible that the symmetric representation of the thalamocortical connections is, by itself, relevant to the efficient functioning of the somatosensory system. Complex sensorimotor processes, such as the currently assessed motor hand skill and whole-body stability, require efficient functioning of a highly specialized and wide neuronal network of the various, if not all, brain areas. This view is supported by the fact that stronger correlations were seen in the asymmetry rather than limb-specific tract values. In addition, it has been shown that despite the unilateral injury, also the non-dominant performance can be affected in hemiplegic CP (Mailleux et al. 2020a).

Most of the correlations were found between the thalamocortical tract AFD values and sensorimotor performance. While no previous studies have reported correlations between the AFD values and sensorimotor performance, it remains to be clarified if the metric is as functionally relevant as it seems. Nevertheless, the observed correlation directions appeared reasonable as the more “typical” AFD values were related to better sensorimotor performance in the patients. Finally, it is important to note that in CP, the damaged sensorimotor pathways or the underdevelopment of interhemispheric connections might restrict the normal hemispheric specialization processes (Ferre et al. 2020). Thus, the same assumptions concerning the structure–function associations are not necessarily relevant in both typical development and in CP.

### Limitations

The sample size (TD = 19, HP = 15, DP = 10) of this study was sufficient to observe significant alterations in the thalamocortical tracts but was still limited. The number of patients in the fMRI-seeded tractography analysis (HP = 8–9, DP = 5) was low but provided nevertheless promising results for future use. Although the statistical power could have been increased by pooling the results of all children with CP together, we chose to keep the hemiplegic and diplegic groups separated due to the distinct topography of their dysfunction. The analysis did not, however, take the lesion type and timing into account. As expected from the literature, the participants with diplegic CP were most often associated with periventricular white matter injury (occurring typically in the early third trimester) whereas half of the participants with hemiplegic CP had gray matter injury (occurring typically later in pregnancy). While thalamocortical tracts are yet to reach their cortical destinations before third trimester, the exact lesion timings can influence the degree of thalamocortical tract involvement greatly. While different lesion types are known to associate with GMFCS levels (Arnfield et al. 2013; Franki et al. 2020) and upper-limb dysfunction (Fiori et al. 2015; Gupta et al. 2017; Mailleux et al. 2017), they can in part influence the results. Future studies would thus benefit from larger sample sizes or stricter inclusion criteria for the injury type allowing the investigation of the effect of the injury type.

The tractography pipeline included manual work, which is subject to intra-rater and inter-rater variability. The primary fMRI activation areas were selected by author TN, the placement of manual-ROIs and the projection of fMRI–ROIs on white matter surface were performed by author JJ and spurious streamlines were cleaned out by author Dogu Baran Aydogan (DBA). Multiple raters at each step might have been beneficial, despite the authors’ experience in their selected responsibilities.

A limiting factor of the study was the decision to compare the dominant and non-dominant thalamocortical tracts. As discussed, some observations can be driven by left–right lateralization rather than dominancy. To overcome this problem, Papadelis et al. (2018) assigned the hemispheres of the control group randomly to be compared against the affected and non-affected hemispheres of the hemiplegic CP group. However, this is not as straightforward with the diplegic participants, and such an approach would lose the interesting aspects of lateralization differences between CP and controls. To develop a full picture of thalamocortical injuries in CP, additional studies with handedness, and lateralization in healthy adolescents will be needed.

Finally, the interpretation of structure–function relationship was restricted by the use of single sum variables for the upper and lower extremities. It can be assumed that especially children with hemiplegic CP could have benefitted from separate analyses for the dominant and non-dominant hand function. However, due to the limited statistical power, it was justified to restrict the analysis to a single hand skill value. The selected performance measures for upper and lower limbs provided satisfactory distributions both within and between the CP and control participants, which is not always possible with typical clinical measures.

## Conclusions

We examined how the thalamocortical pathways are affected in hemiplegic and diplegic spastic CP. For the first time, these thalamocortical tracts were compared separately for the upper and lower extremities, and their association with sensorimotor performance was evaluated. We showed that children with hemiplegic CP had less symmetrically affected white matter structure than those with diplegic CP in terms of MD. In both CP groups, the structural deficits seemed similar for the tracts projecting to upper or lower limb cortical representation areas. The manual “anatomical” and fMRI-guided “functional” seeding approaches yielded comparable results of the thalamocortical tract properties, therefore indicating the potential of our novel proprioception-based fMRI-seeding approach in the future tractography studies. Overall, our results suggested that the research targeted to limb-specific thalamocortical tracts may help to better understand the relationship between white matter structure and specific sensorimotor performance in CP as well as in typical development.

## Supplementary Material

SupplementaryMaterial_final_bhad279

## Data Availability

The data that support the findings of this study are available on request from the corresponding author.
