# Supplementary material for: Limb-specific thalamocortical tracts are impaired differently in hemiplegic and diplegic subtypes of cerebral palsy
Source: Cereb Cortex. 2023 Aug 18;33(19):10245–57. doi: 10.1093/cercor/bhad279 (PMC10545439; doi:10.1093/cercor/bhad279)
Supplement: SupplementaryMaterial_final_bhad279 [file supplementarymaterial_final_bhad279.docx]

# Supplementary material

**Table S1.** Summary of the studied participants.

|  | Hemiplegic cerebral palsy | Diplegic cerebral palsy | Typically developed |
| --- | --- | --- | --- |
| N (dMRI) | 15 | 10 | 19 |
| Sex (male / female) | 4 / 11 | 5 / 5 | 5 / 14 |
| Age (mean ± std) | 13.7 ± 2.2 | 13.4 ± 2.0 | 14.2 ± 2.5 |
| Dominancy (left / right) | 9 / 6 | 4 / 6 | 0 / 19 |
| GMFCS (I / II) | 14 / 1 | 3 / 7 | - |
| MACS (I / II / III / unknown) | 6 / 6 / 1 / 2 | 5 / 1 / 1 / 3 | - |
| Injury type (grey matter / white matter / other^a^) | 7 / 6 / 2 | 0 / 6 / 4 | - |
| Preterm (yes / no / unknown) | 5 / 8 / 2 | 5 / 1 / 4 | - |

^a^ maldevelopment, miscellaneous or normal
GMFCS = Gross motor function classification system; MACS = Manual ability classification system

**Table S2.** Sensorimotor tests results (mean ± standard deviation).

|  | Hemiplegic cerebral palsy | Diplegic cerebral palsy | Typically developed |
| --- | --- | --- | --- |
| **Nine-Hole-Peg** (performance time, s) |  |  |  |
| N (of which imputation was used) | 13 (2) | 7 (1) | 16 |
| Dominant hand | 19.4 ± 3.3 | 20.0 ± 1.2 | 16.7 ± 1.9 |
| Non-dominant hand | 57.5 ± 37.9 | 24.2 ± 2.7 | 19.1 ± 2.6 |
| **Box-and-Block** (blocks moved / 60s) |  |  |  |
| N (of which imputation was used) | 13 | 7 | 16 |
| Dominant hand | 66.8 ± 12.1 | 60.0 ± 7.6 | 72.1 ± 8.1 |
| Non-dominant hand | 39.3 ± 15.9 | 50.4 ± 16.1 | 70.3 ± 6.3 |
| **Standing stability** (velocity, mm/s) ^a^ |  |  |  |
| N (of which imputation was used) | 15 | 9 (2) | 17 (1) |
| Normal stand, eyes open | 8.6 ± 2.6 | 11.3 ± 4.7 | 6.4 ± 3.0 |
| Normal stand, eyes closed | 9.0 ± 2.3 | 11.7 ± 4.8 | 7.0 ± 2.5 |
| Feet together, eyes open | 12.3 ± 3.3 | 12.1 ± 2.8 | 8.5 ± 3.2 |
| Feet together, eyes closed | 18.0 ± 9.3 | 14.3 ± 6.2 | 12.8 ± 4.2 |
| **Gait stability** (complexity, RCME) ^a^ |  |  |  |
| N (of which imputation was used) | 15 | 9 | 17 (1) |
| Vertical RCME normal | 0.1 ± 0.8 | 0.9 ± 1.2 | −0.5 ± 0.5 |
| Vertical RCME motor | 0.1 ± 0.8 | 1.2 ± 1.5 | −0.5 ± 0.5 |
| Vertical RCME cognitive | 0.3 ± 0.9 | 1.3 ± 1.6 | −0.4 ± 0.5 |
| Horizontal RCME normal | 0.3 ± 0.9 | 0.4 ± 0.9 | −0.6 ± 0.9 |
| Horizontal RCME motor | 0.5 ± 0.9 | 0.6 ± 1.0 | −0.4 ± 1.0 |
| Horizontal RCME cognitive | 0.3 ± 1.0 | 0.6 ± 0.9 | −0.8 ± 0.8 |

^a^ The experimental setup and group differences have been previously described by Piitulainen et al. (2021).
RCME = refined-compound-multiscale entropy (Ihlen et al., 2016; obtained from inertial-measurement-unit)


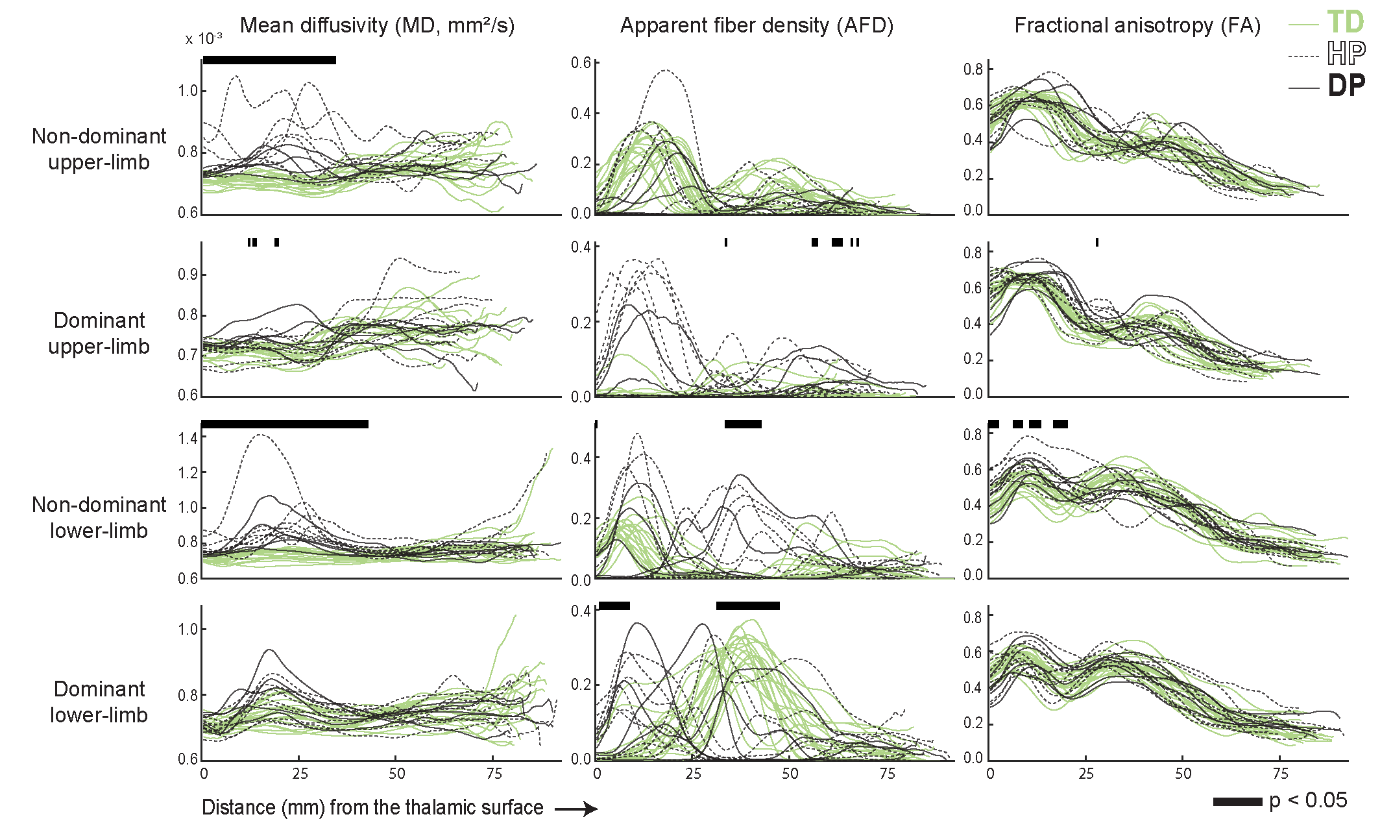


**Figure S1.** Mean diffusivity (MD), apparent fiber density (AFD) and fractional anisotropy (FA) values along the fMRI-seeded thalamocortical tracts. Black lines on top of each graph indicate the positions where the three groups differ significantly (p < 0.05) from each other. For illustration, we used smoothing with 10-point moving average. Position 0 is located on thalamus surface. TD = typically developed, HP = hemiplegia, DP = diplegia


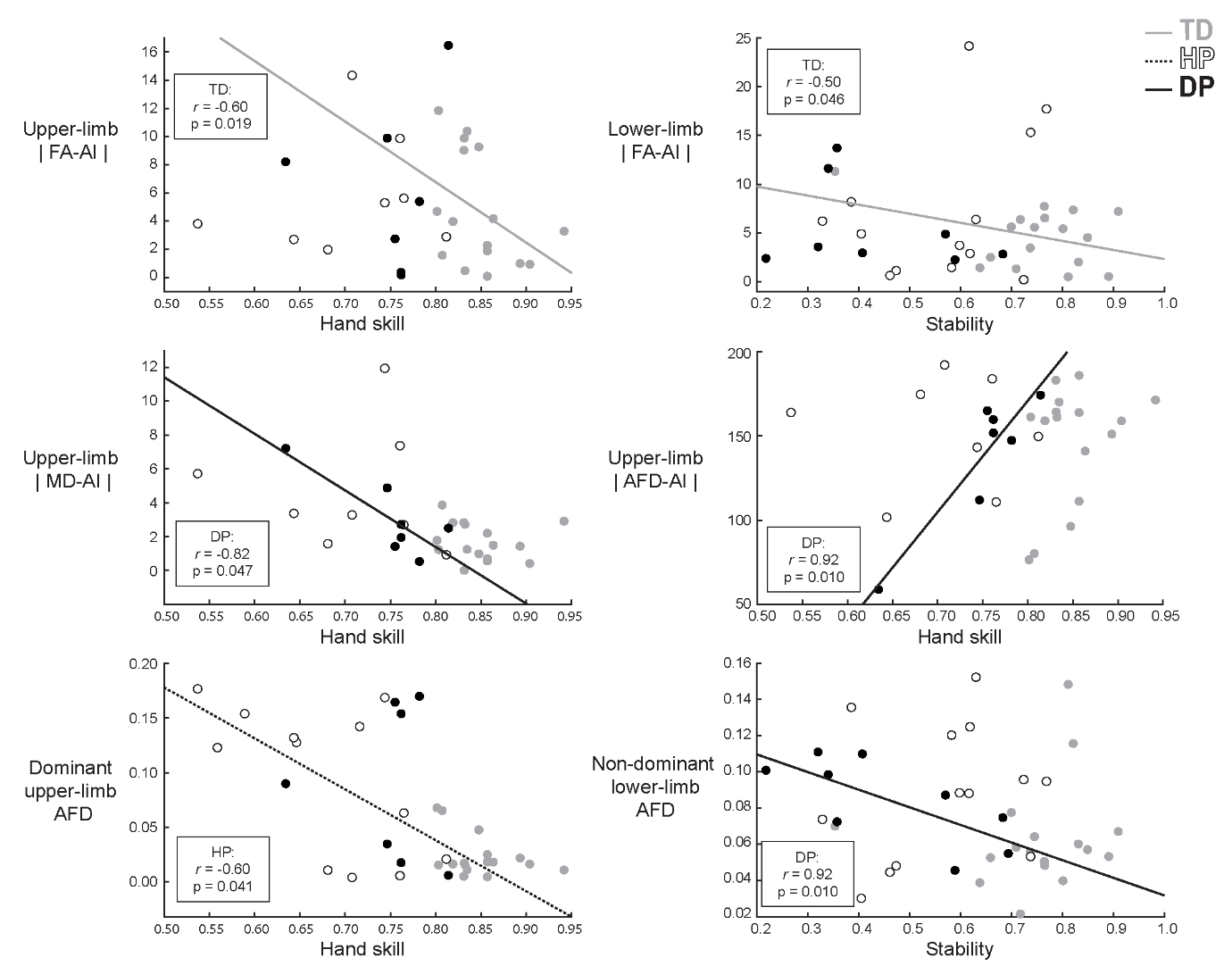


**Figure S2.** Examples of scatter plots between manual-seeded thalamocortical tract properties and sensorimotor performance. Groupwise correlations with p < 0.05 are indicated with least square fitted lines. Please note that in the statistical analysis the correlations were corrected for age (results shown in boxes).
